# CD19-TANK细胞治疗复发/难治B细胞淋巴瘤15例临床观察

**DOI:** 10.3760/cma.j.issn.0253-2727.2022.02.012

**Published:** 2022-02

**Authors:** 旭东 张, 晓瑞 付, 振昌 孙, 蕾 张, 鑫 李, 玲 李, 晶晶 吴, 新华 王, 飞飞 南, 宇 常, 慧 于, 兆明 李, 明智 张

**Affiliations:** 郑州大学第一附属医院肿瘤科，郑州 450052 Department of Oncology, The First Affiliated Hospital, Zhengzhou University, Zhengzhou 450052, China

B细胞淋巴瘤是淋巴瘤中最常见的类型，其中发病率最高的为弥漫大B细胞淋巴瘤（DLBCL），其次为套细胞淋巴瘤（MCL）[1]–[2]。近二十年来利妥昔单抗显著改善B细胞淋巴瘤患者预后，但仍有近30％患者因复发、难治无法获益[3]。过继性细胞免疫治疗尤其是CD19嵌合抗原受体（CAR）T细胞（CAR-T细胞）在复发/难治B细胞淋巴瘤中取得了显著疗效，但制备复杂、存在脱靶效应、治疗费用高及严重的并发症等原因限制了CAR-T细胞在临床广泛应用[4]–[5]。NK细胞目前认为是除T细胞外另一种重要的工程细胞。CAR-NK细胞治疗由于不良反应少、生产高效[6]，近年来在免疫治疗中越来越受到重视。

CD19靶向活化自然杀伤细胞（CD19-TANK细胞）主要应用于CD19阳性B细胞淋巴瘤的治疗。CD19抗原在不同阶段的B细胞淋巴瘤中均有表达，且表达率高达95％，因此CD19-TANK细胞可以靶向绝大多数B细胞淋巴瘤[7]。本研究中，我们初步观察和分析了CD19-TANK细胞治疗复发/难治B细胞淋巴瘤的疗效与安全性，总结如下。

## 病例与方法

1. 病例资料：分析2016年5月至2018年10月在郑州大学第一附属医院肿瘤科接受CD19-TANK细胞治疗的15例复发/难治B细胞淋巴瘤患者，男13例，女2例，中位年龄55（25～81）岁。其中DLBCL 12例，MCL 3例。6例为复发病例，9例为难治病例。将患者分为两组，其中例1～7接受CD19-TANK细胞单药治疗，例8～15接受CD19-TANK细胞联合化疗/免疫药物治疗，治疗前临床资料见表1。入组标准：①病理学明确诊断为B细胞淋巴瘤；②前期均经过多线或多周期化疗及免疫治疗方案；③25～85岁；④CD19阳性；⑤充分了解本研究且签署知情同意书，依从性较好，可持续随访。出组标准：①CD19-TANK细胞治疗过程中出现危及生命的不良事件；②未能定期复查。本研究获得我院临床试验伦理委员会批准（批件号：2021-KY-0725-003），所有病例或其家属均充分知情同意并签署知情同意书。

**表1:** 15例复发/难治B细胞淋巴瘤患者CD19靶向活化自然杀伤细胞（CD19 TANK细胞）治疗前临床资料

序号	性别	年龄（岁）	疾病	分期	IPI评分	前期治疗［方案（疗程数）］
1	女	27	DLBCL	Ⅳ期	1	R-CHOP（4）
2	男	30	DLBCL	Ⅳ期	3	R-CHOP（6）+EPOCH（2）+放疗+ESHAP（2）
3	男	38	DLBCL	Ⅳ期	3	CHOP（2）+CVP（2）+GDP（1）+放疗+R-ESHAP（4）+EPOCH（4）
4	男	62	MCL	Ⅳ期	2	CHOP（2）+EPOCH（6）
5	男	57	DLBCL	Ⅳ期	1	CHOP（5）+ESHAP（2）+EPOCH（2）+放疗
6	男	64	MCL	Ⅳ期	3	R-CHOP（3）+R-DHAP（3）+R-DICE（1）+GEMOX（1）+DHAP（2）+CHOP（1）+R-FC（1）
7	男	81	DLBCL	Ⅳ期	3	R-CVP（6）
8	男	45	DLBCL	ⅣB期	2	R-EPOCH（2）+R-CHOP（2）+EPOCH（2）
9	男	32	DLBCL	Ⅳ期	4	CHOP（6）+MA（1）+（R+DXM+PEM+FTM）（1）
10	男	40	DLBCL	Ⅳ期	2	EPOCH（17）
11	男	59	MCL	Ⅳ期	2	CHOP（2）+DHAP（4）+FC（2）
12	男	55	DLBCL	ⅡE期	1	CHOP（6）+ESHAP（2）
13	男	60	DLBCL	Ⅲ期	2	R-CODOX-M（2）+R-IVAC（1）+R-EPOCH（3）+GDPT（2）
14	女	25	DLBCL	ⅣB期	3	CHOP（2）+EPOCH（5）+DICE（1）+MINE（1）+GDPT（1）
15	男	65	DLBCL	Ⅲ期	4	CHOP（8）+ESHAP（7）+GDP（1）+GEMOX（1）+放疗

注：DLBCL：弥漫大B细胞淋巴瘤；IPI：国际预后指数；R：利妥昔单抗；CHOP：环磷酰胺+长春新碱+表柔比星+泼尼松；EPOCH：依托泊苷+多柔比星+长春新碱+泼尼松+环磷酰胺；ESHAP：依托泊苷+甲泼尼龙+顺铂+阿糖胞苷；CVP：环磷酰胺+长春新碱+泼尼松；GDP：吉西他滨+顺铂+地塞米松；DHAP：顺铂+阿糖胞苷+地塞米松；DICE：地塞米松+异环磷酰胺+卡铂+依托泊苷；GEMOX：吉西他滨+奥沙利铂；FC：氟尿嘧啶+顺铂；MA：米托蒽醌+阿糖胞苷；DXM+PEM+FTM：地塞米松+培美曲塞+福莫司汀；CODOX-M：环磷酰胺+长春新碱+阿霉素+大剂量甲氨蝶呤；IVAC：异环磷酰胺+依托泊苷+阿糖胞苷；GDPT：吉西他滨+顺铂+波尼松+沙利度胺；MINE：异环磷酰胺+米托蒽醌+依托泊苷

2. CD19-TANK细胞来源：本项目所用CD19-TANK细胞为宜明昂科生物医药技术（上海）有限公司自主研发的细胞免疫治疗产品。

3. 疗效评价：单用CD19-TANK细胞治疗组每7 d治疗1次，每次输注1×10^9^细胞量，28 d为1个周期；化疗后序贯细胞治疗组患者化疗结束后7 d开始应用CD19-TANK细胞治疗，每次输注1×10^9^细胞量，每7 d治疗1次，28 d为1个周期。例6在输注2次后病情进展，停用CD19-TANK细胞治疗；例9共治疗3次，3次后评价疗效，其余患者均在完成两个治疗周期后进行疗效评价。观察骨髓细胞形态学缓解情况，采用流式细胞术、增强CT/MRI或PET-CT检测残留病变。疗效评价参考2016年NCCN推荐的Lugano淋巴瘤疗效标准。

4. 不良反应：输注CD19-TANK细胞期间监测血压、呼吸、心率等生命体征，每次输注细胞结束后3 d复查血常规、生化等指标，参照WHO化疗药物毒性反应分度标准进行不良事件分级，主要记录骨髓抑制、过敏反应等不良反应发生情况。

## 结果

单用CD19-TANK治疗组7例患者中，完全缓解（CR）1例，疾病稳定（SD）4例，疾病进展（PD）2例。除例5在输注CD19-TANK期间有轻度低热，余未观察到明显的不良反应。8例化疗序贯CD19-TANK细胞组患者，部分缓解（PR）1例，SD 5例，PD 2例。观察到骨髓抑制等不良反应，多为化疗所致，无证据表明细胞治疗增加化疗不良反应。各病例具体转归见图1。全部15例患者中，单用CD19-TANK治疗组中位无进展生存（PFS）与总生存（OS）时间分别为48（95％ *CI* 14～76）个月、55（95％ *CI* 6～67）个月，联合用药组中位PFS与OS时间分别为11（95％ *CI* 3～16）个月、14（95％ *CI* 0.5～15）个月，提示单用CD19-TANK组患者的生存预后明显优于联合用药组（*P*值分别为0.009、0.042）。但由于患者2个周期疗效评估SD或PD可自行选择其他挽救治疗手段，因此生存获益不是完全因CD19-TANK细胞所致。

**图1:**
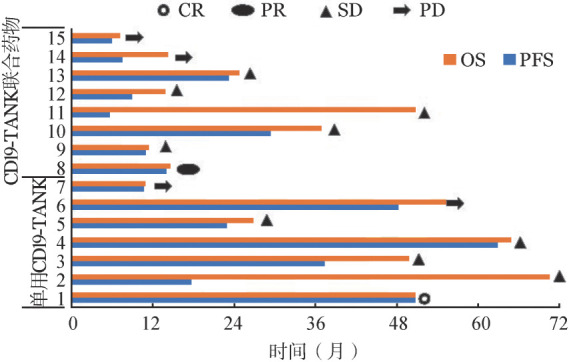
15例复发/难治B细胞淋巴瘤患者CD19靶向活化自然杀伤细胞（CD19-TANK细胞）治疗后疗效与转归 OS:总生存；PFS：无进展生存；CR：完全缓解；PR：部分缓解；SD：疾病稳定；PD：疾病进展

## 讨论

NK细胞过继性免疫治疗是细胞生物治疗的重要手段，在抗肿瘤方面发挥重要的作用[8]。CAR-NK细胞的抗肿瘤作用在实体瘤、血液系统恶性肿瘤中的临床前研究中得到了越来越多的验证[9]–[11]，具有更好安全性、更强天然抗肿瘤活性和更大“通用型”潜力等优势[12]。目前已有十余个CD19-CAR-NK细胞治疗白血病/淋巴瘤的临床试验在进行中，初步结果提示安全性显著优于CAR-T治疗，靶点涵盖了CD19、CD22、BCMA、CD33等[13]。目前CAR-NK虽显示了不同程度的抗肿瘤作用，但不同类型、来源和靶点的CAR-NK细胞疗效具有较明显异质性[14]，个体差异也较大[15]–[16]。

本组15例复发/难治B细胞淋巴瘤患者单用或联合免疫化疗整体的有效率均未到15％，低于文献[17]报道常见的二线挽救治疗效果。考虑可能和入组患者前期治疗和体力状态有关。大部分患者均经历了超过两种化疗方案、8个周期以上化疗，对化疗敏感性已严重降低，或本身即存在较强的多药耐药情况；另外，患者体力状态和化疗意愿较差，部分患者已不能耐受正常剂量的化疗，需要停药或减量治疗；更重要的是，很多患者具有较高肿瘤负荷，甚至大肿块情况，导致CD19-TANK细胞很难穿透肿瘤组织，有效分布肿瘤微环境。而两例获得CR和PR的患者，前期接受的化疗方案较少［分别为1种方案（CHOP）和2种方案（CHOP和EPOCH）］，化疗周期较短（分别为4个周期和6个周期），且肿瘤负荷较小。其中例1是肠道起源的B细胞淋巴瘤，腹痛为主要症状，复发两次，急诊入院手术。前期治疗过程中患者不能耐受免疫化疗。后采用CD19-TANK细胞单独输注1次后，腹痛症状即得到改善，2个周期后疗效评价达到CR，随后巩固输注1次，输注期间，除了轻微发热，无其他不适，期间也未接受任何形式的其他治疗，现无进展生存14个月。

另外，联合治疗患者也仅有1例达到PR，单用CD19-TANK患者显示了更好的生存获益，提示TANK细胞治疗和化疗可能无协同作用，对于经历多线多周期化疗复发难治B细胞患者，常规化疗作用有限，需要如免疫治疗、靶向治疗等低毒高效治疗手段，改善其预后和生活质量。本研究因病例数少，且前期治疗情况、个体耐受性、治疗意愿等差异性较大，两组患者的生存差异可能并不完全是TANK细胞作用，本文仅做描述性分析，后期仍需更多随机对照研究，以及药物动力学和代谢学证据。目前如何提高CAR-NK的疗效的研究，多集中于靶点、共刺激信号和协同靶点的选择，例如利用趋化因子受体CXCR4的进一步修饰、TGF-β嵌合受体或联合靶向EGFR等加强CAR-NK细胞的活性和杀伤作用，以及克服肿瘤灌注不良、免疫抑制微环境等[18]。另外，由于NK细胞本身极少表达或分泌PD-1，几乎不会诱导免疫抑制，理论上也不会影响PD-1抗体效价[19]。因此，有研究提示CAR-NK和PD-1抗体联合治疗的潜力更好。

CAR-T细胞治疗常见的不良反应有细胞因子释放综合征（CRS）和脑病综合征，这两种并发症是严重甚至是致命的[20]。本研究中，我们发现CD19-TANK除了轻微的输注反应、轻度发热外，未见其他短期或长期不良反应。这和目前已有研究结果一致，NCT02944162临床试验中一次注射剂量升至5×10^9^仅引起少量患者的轻微发热和CRS[21]。理论上讲，NK细胞几乎不分泌INF-γ、TNF-α、IL-1、IL-6等容易引起CRS的细胞因子，分泌的IL-3和GM-CSF等很少会引起CRS[22]。自体CAR-T细胞由于其制作程序复杂，且花费时间较长，对一些病情进展迅速的患者来说是不适合的，异基因CAR-T细胞治疗发生移植物抗宿主病（GVHD）的可能性较高。NK细胞靶向肿瘤细胞不需要抗原提呈或HLA相合，异基因NK细胞几乎不会产生GVHD[23]。

在所有患者中，有研究显示调节性T细胞和髓系来源细胞对CAR-NK的细胞活性有负面调控作用[24]。和CAR-T细胞不同，TANK细胞的抗肿瘤活性特异性识别肿瘤抗原的CAR有关，还与NK细胞自身受体有关，特别是刺激信号和抑制信号之间的平衡，这些信号激活并释放了穿孔素和颗粒酶[25]，往往决定了NK细胞的活性[26]；抗体依赖细胞介导的细胞毒作用也是NK细胞的另外一个杀伤机制，TANK细胞对CD19阴性或丢失的淋巴瘤细胞仍有杀伤作用。因此，我们考虑该患者可能自身NK细胞受体在CAR-NK细胞刺激下，激活或释放了更多有助于提高NK细胞抗肿瘤活性的细胞因子。目前，研究人员也试图通过各种方法改善CAR-NK治疗疗效，以及筛选潜在的获益人群。例如，构建更理想的人源化动物（PDX）模型、免疫模型等。减少脱靶效应，寻找更适合NK细胞或NK-92细胞的CAR相关受体[5]，改良载体转导NK细胞效率[27]，增加NK细胞杀伤活性[28]等。
